# Proteomic Insights into Senescence of Testicular Peritubular Cells from a Nonhuman Primate Model

**DOI:** 10.3390/cells9112498

**Published:** 2020-11-17

**Authors:** Jan B. Stöckl, Nina Schmid, Florian Flenkenthaler, Charis Drummer, Rüdiger Behr, Artur Mayerhofer, Georg J. Arnold, Thomas Fröhlich

**Affiliations:** 1 Laboratory for Functional Genome Analysis LAFUGA, Gene Center, LMU München, 81377 Munich, Germany; stoeckl@genzentrum.lmu.de (J.B.S.); flenkenthaler@genzentrum.lmu.de (F.F.); 2 LMU München, Biomedical Center (BMC), Anatomy III—Cell Biology, 82152 Martinsried, Germany; Nina.Schmid@lrz.uni-muenchen.de (N.S.); mayerhofer@lrz.uni-muenchen.de (A.M.); 3 Platform Degenerative Diseases, German Primate Center, Leibniz Institute for Primate Research, 37077 Göttingen, Germany; cdrummer@dpz.eu (C.D.); rbehr@dpz.eu (R.B.); 4 DZHK (German Center for Cardiovascular Research), Partner Site Göttingen, 37077 Göttingen, Germany

**Keywords:** aging, testis, cellular model, proteome, marmoset monkey, nonhuman primate, senescence, secretome

## Abstract

Age-related changes in the human testis may include morphological alterations, disturbed steroidogenesis, and impaired spermatogenesis. However, the specific impact of cell age remains poorly understood and difficult to assess. Testicular peritubular cells fulfill essential functions, including sperm transport, contributions to the spermatogonial stem cell niche, and paracrine interactions within the testis. To study their role in age-associated decline of testicular functions, we performed comprehensive proteome and secretome analyses of repeatedly passaged peritubular cells from *Callithrix jacchus*. This nonhuman primate model better reflects the human testicular biology than rodents and further gives access to young donors unavailable from humans. Among 5095 identified proteins, 583 were differentially abundant between samples with low and high passage numbers. The alterations indicate a reduced ability of senescent peritubular cells to contract and secrete proteins, as well as disturbances in nuclear factor (NF)-κB signaling and a reduced capacity to handle reactive oxygen species. Since this in vitro model may not exactly mirror all molecular aspects of in vivo aging, we investigated the proteomes and secretomes of testicular peritubular cells from young and old donors. Even though the age-related alterations at the protein level were less pronounced, we found evidence for impaired protein secretion, altered NF-κB signaling, and reduced contractility of these in vivo aged peritubular cells.

## 1. Introduction

Aging, in general, is associated with a loss of cellular and tissue integrity and finally leads to irreversibly impaired organ functions [1]. In addition to many other tissues and organs, there is some evidence that human testes are affected by age [2,3,4]. Typical signs of testicular aging were reported to include reduced androgen production, increased de novo mutations in the germ cells, impaired spermatogenesis, and decreased sperm quality [5]. Furthermore, structural changes including thickening of the tubular walls were reported; however, there are also reports implying that these structural changes do not necessarily have an impact on fertility [6].

Although human testicular peritubular cells (HTPCs) have been the focus of functional studies recently and provide a window into the human testis, these testicular cells are still the least explored cell type of the male gonad [7]. They can be isolated from routine biopsies collected, e.g., during re-ligation of the vas deferens from vasectomized men or during surgery performed for other reasons. HTPCs can readily be cultured for several passages, a fact facilitating in vitro studies [8]. In man, testicular peritubular cells in situ form several layers of cells, which together with the extracellular matrix (ECM) surround the walls of seminiferous tubules. These myofibroblastic cells provide structural support, contract, and are thought to transport sperm. They further secrete, for example, extracellular matrix proteins. In this context, HTPCs and their role in the maintenance of the extracellular matrix (ECM), as well as their contribution to testicular signaling, were shown to be crucial in spermatogenesis [9,10,11]. They also produce important growth factors, e.g., glial cell line-derived neurotrophic factor (GDNF) and stromal cell-derived factor 1 (CXCL12) [12,13]. GDNF deletion in TPCs disrupts spermatogonia development, indicating an essential role of TPCs in maintaining the spermatogonial stem cell (SSC) niche [14]. Further data indicate that TPCs are important mediators and executors of androgen signaling, since a TPC-specific knockout of AR in mouse testis showed decreased sperm count and testis size, detrimental effects on Sertoli cells’ ability to maintain germ cells, and defective ultrastructure of Leydig cells [15]. An additional role of TPCs in the testis is the secretion of pigment epithelium-derived factor (SERPINF1), a strong antiangiogenic factor [16,17,18,19]. Hence, TPCs have important roles in the testis and the possibility to study them in vitro makes them a highly interesting and relevant target to extend the study to their roles in testicular aging.

Models to study aging-related changes include replicative senescence [20]. Senescent cells are typically cell-cycle-arrested, and telomere attrition is one of several key mechanisms for triggering cellular senescence [1,21,22,23,24]. Senescent cells undergo typical alterations in morphology and shape, increase in size, and develop a senescence-associated secretory phenotype (SASP). A pronounced expression of senescence-associated beta-galactosidase is a widely used marker for cellular senescence [25,26,27]. Repeated cell passaging is commonly employed to trigger senescence in various cell types [21,28,29]. It has been demonstrated that this approach also induces cellular senescence in HTPCs, leading to increases in cell size, to telomere attrition, and to an SASP. Furthermore, a reduced mitochondrial network and an increased lysosome count was detected by focused ion beam scanning electron microscopy (FIB/SEM) tomography. Comprehensive proteome and secretome datasets were generated, which revealed several proteins previously not known to be involved in aging [30].

When studying human cells, it remains impossible to evaluate the sole impact of age on cells, because of confounding factors, such as medical conditions, previous medications, and lifestyle. Furthermore, although triggering senescence in vitro is an established method to study cellular senescence, the results have to be validated in vivo in order to show their relevance for translational research. To achieve this, a comparison of cells from young and old individuals is mandatory. Obtaining a testicular tissue sample solely for research purposes from young healthy men is unethical and not possible. Therefore, a surrogate nonhuman primate model has previously been established, and the relevance of this model for the human system has been demonstrated [31]. We reasoned that this model, i.e., cultured TPCs from the common marmoset (termed MKTPC), may facilitate the investigation of cellular senescence triggered in vitro, as well as in vivo studies by comparison of primary TPCs from young and old individuals. To characterize senescence and age-dependent alterations in the MTKPC model, we chose a mass-spectrometry-based proteomics approach. Such an approach was also the method of choice in studies of human TPCs and, hence, the results from nonhuman primates can be readily compared with those from human [30].

## 2. Materials and Methods

### 2.1. Animals

Common marmoset monkeys (*Callithrix jacchus*) were from the self-sustaining marmoset monkey colony of the German Primate Center (Deutsches Primatenzentrum; DPZ, Leibniz Institute for Primate Research, Göttingen). All animals used for this study were between 2 and 15 years old, i.e., ranging from young adult, sexually mature healthy animals to aged animals [32,33]. Marmoset monkey testes were obtained from animals euthanized for scientific purposes unrelated to this study or castrated for colony management purposes. Euthanasia and castration were performed by experienced veterinarians.

### 2.2. Cell Culture

Tissue samples, which were used for MKTPC isolation, stemmed from the self-sustaining marmoset monkey colony of the German Primate Center. Organ removal from the marmoset monkeys was executed in compliance with crucial institutional guidelines and legal regulations, the German Animal Protection Act (§ 7 Abs. 2 Nr. 3, Tierschutzgesetz). The MKTPCs were isolated and cultured, as described previously [8,31]. In brief, the cells grow on 0.1% gelatin-coated cell culture dishes and were incubated at 37 °C/5% CO_2_ in Dulbecco’s modified Eagle’s medium (DMEM) high glucose (Gibco, Paisley, UK) with supplementary 10% fetal calf serum (Capricom Scientific GmbH, Ebersdorfergrund, Germany) and 1% penicillin/streptomycin (Life Technologies, Carlsbad, CA, USA). Cells derived from six young adult, healthy donors (2 years (yrs) 3 month (mo), 3 yrs, 3 yrs 10 mo, 2 yrs 8 mo, 2 yrs 2 mo, and 2 yrs 9 mo) and from additional six old, healthy animals (11 yrs 4 mo, 9 yrs 1 m, 11 yrs 8 mo, 10 yrs 2 mo, 15 yrs, and 11 yrs 7 mo) were used for the study. Animals at the age of 8 years or older can be considered aged [33].

The MKTPC samples for mass spectrometry analysis were seeded onto cell culture dishes (60 × 15 mm, 21 cm^2^; Sarstedt, Nümbrecht, Germany) and grown under the conditions described above. At a confluency of 90%, the cells were washed with 2 mL of medium without FCS and phenol red; then, 2 mL of fresh medium (without FCS or phenol red) was added. After 24 h, the conditioned medium was removed, centrifuged (3 min, 1000× *g*), and stored at −80 °C until preparation for mass spectrometry analysis. Serial passaging of MKTPCs was implemented until signs of senescence appeared (increased cell size, β-galactosidase expression). The cells were harvested (trypsinized, washed five times with PBS) and likewise stored until collective analysis.

### 2.3. Cell Size Measurement

The mean diameter of trypsinized MKTPCs was measured using an automated cell counting device (CASY system, Schärfe Systems, Reutlingen, Germany) as described earlier [34].

### 2.4. β-Galactosidase Staining

Senescence-associated β-galactosidase staining was carried out using a commercial kit (Senescence β-Galactosidase Staining Kit, Cell Signaling Technology, Danvers, MA, USA). Cultured MKTPCs were seeded onto cover slips, and the staining was implemented according to manufacturer’s instructions. A Zeiss Axiovert microscope (Zeiss GmbH, Oberkochen, Germany) was used for the examination.

### 2.5. Sample Preparation for Mass Spectrometry Analysis

Cells were lysed in 8 M urea in 50 mM ammonium bicarbonate and sonicated with a cup resonator (Bandelin). Samples were homogenized with centrifugation devices (Qiashredder, Qiagen (2500× *g*, 2 min)). Protein content was measured with a Pierce 660 nm assay (Thermo). Proteins were reduced in 4 mM dithiothreitol/2 mM tris(2-carboxyethyl)phosphine and alkylated in 8 mM iodoacetamide. Digestion was performed with Lys-C (1/100, enzyme/protein, Wako) for 4 h followed by trypsin (1/50 enzyme/protein, Promega) overnight at 37 °C. For the secretome analysis, cell supernatants were concentrated using 3 K Amicon centrifugal filters (Merck). Samples were brought to a concentration of 1 M urea in 50 mM ammonium bicarbonate. Afterward, samples were treated in the same way as the cell lysates apart from the digestion, which was done in a single step using a Lys-C/Trypsin Mix (1/25 enzyme/protein, Promega) overnight at 37 °C.

### 2.6. Liquid Chromatography–Tandem Mass Spectrometry Analysis

LC–MS/MS analysis was performed using an Ultimate 3000 RSLC connected to a Q Exactive HF-X mass spectrometer. From cell lysates and secretomes, 2 µg and 1 µg of total protein were injected, respectively. Samples were first loaded onto a trap column (PepMap 100 C18, 100 µm × 2 cm, 5 µM particles, Thermo Scientific) using a flow rate of 5 µL/min (mobile phase: 0.1% formic acid and 1% acetonitrile in water). Liquid chromatography was performed with an EASY-Spray column (PepMap RSLC C18, 75 µm × 50 cm, 2 µm particles, Thermo Scientific) and a flow rate of 250 nL/min. Cell lysate samples were separated with a two-step gradient from 3% B (0.1% formic acid in acetonitrile) to 25% B in 160 min followed by a ramp to 40% for 10 min (A: 0.1% formic acid in water). Using the same eluents, the gradient for secretome samples started from 3% to 25% B in 30 min, followed by ramping to 40% B in 5 min. The mass spectrometer was run in the data-dependent acquisition mode with a maximum of 15 MS/MS spectra per survey scan.

### 2.7. Data Analysis

MaxQuant (1.6.5.0) [35] was used to process LC–MS/MS data. *Callithrix jacchus* protein sequences were retrieved from UniProt (retrieval date: 02/2020) and complemented by the built-in contaminant database. A label-free quantification approach was chosen, and the “match between runs” feature was enabled. For data analysis, Perseus (1.6.5.0) and R (3.6.3) were used [36]. Volcano plots of the pMKTPCs data were generated using modified (s0 = 0.1) paired Welch *t*-tests [37]. Volcano plots for the comparison for old and young MKTPCs were generated with the Perseus built-in volcano plot function with the parameters s0 = 0.1 and false discovery rate (FDR) <0.05 for the calculation of the cutoff curve. Annotation analysis was done with Database for Annotation, Visualization, and Integrated Discovery (DAVID) and Gene Set Enrichment Analysis (GSEA) [38,39,40,41]. REVIGO was used to remove redundant terms from GSEA results [42]. For the differentially abundant proteins of the secretome, SignalP and SecretomeP were used to predict signal peptides and nonclassical protein secretion, respectively [43,44]. The mass spectrometry proteomics data were deposited to the ProteomeXchange Consortium (http://proteomecentral.proteomexchange.org) via the Proteomics Identification Database (PRIDE) partner repository with the dataset identifier PXD022165 [45].

## 3. Results

### 3.1. Proteome Analysis of Cells and Secretomes from Low- vs. High-Passage MKTPCs

**Figure 1:**
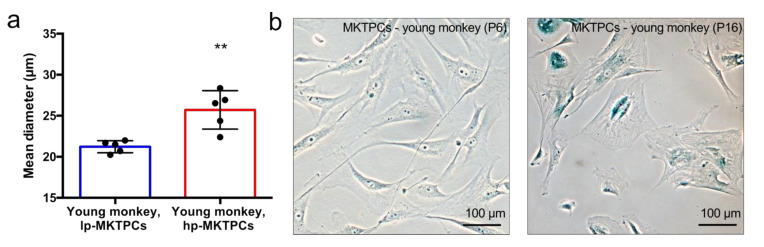
(**a**) Cell size measurement of low-passage-number vs high-passage-number testicular peritubular cells from the common marmoset (lp-MKTPCs vs. hp-MKTPCs) from the same donor animal. A significant increase in cellular diameter of hp-MKTPCs was shown. Columns indicate the mean; bars indicate the standard deviation; for statistics, a paired *t*-test was used. (**b**) Light micrograph of senescence-associated β-galactosidase staining of lp-MKTPCS and hp-MKTPCs. ** *p*-value < 0.005

A proteome analysis of low-passage-number MKTPCs (lp-MKTPCs) and high-passage-number MKTPCs (hp-MKTPCs) was conducted, using MKTPCs derived from five young healthy donors. Secretomes and proteomes were analyzed by LC–MS/MS from cells of the same donors after 2–3 and after 10–12 passages, respectively. MKTPCs proliferated in culture showed, after more than 10 passages, a significant increase in cell size and changes in cell shape, expressed considerable amounts of senescence-associated beta galactosidase, and stopped dividing (Figure 1).

**Figure 2:**
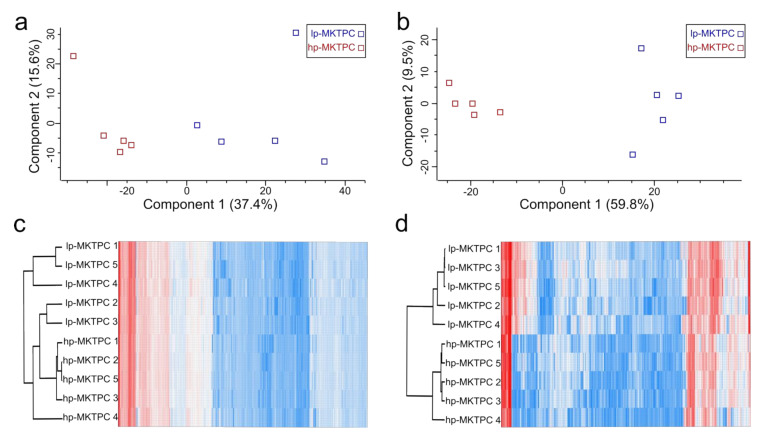
Principal component analysis (PCA) of cellular proteomes (**a**) and secretomes (**b**) of MKTPCs derived from lp-MKTPCs and hp-MKTPCs; each square represents an individual donor. Heat map and unsupervised hierarchical clustering of cellular proteomes (**c**) and secretome (**d**).

In total, 4916 and 1132 proteins and 66, 240 and 8786 peptides were identified from the proteome and secretome, respectively (Tables S1 and S2, Supplementary Materials). Typical testicular peritubular cell markers, namely, calponin 1 (CNN1), smooth muscle actin (ACTA2), and myosin heavy chain 11 (MYH11), were identified. A principal component analysis (PCA) based on the quantitative protein abundance data showed clear separation of the proteomes and secretomes of hp-MKTPCs and lp-MKTPCs (Figure 2a,b). For both cellular proteome and secretome, unsupervised hierarchical clustering indicated clustering of lp-MKTPCs and hp-MKTPCs (Figure 2c,d).

**Figure 3:**
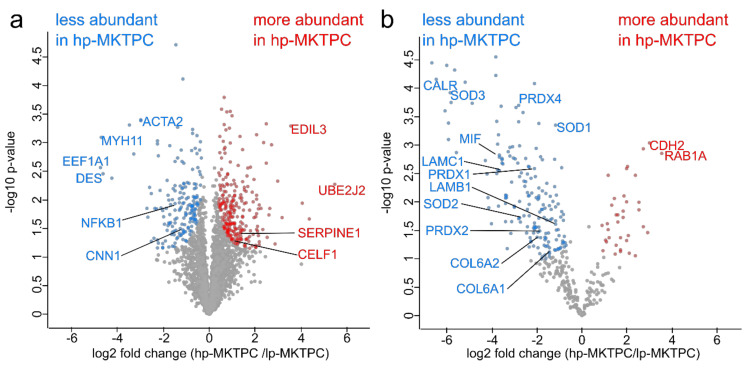
Volcano plots of hp-MKTPC vs. lp-MKTPC proteomes (**a**) and secretomes (**b**). For statistics, a paired Welch *t*-test was used. Results were corrected for multiple testing with a false discovery rate (FDR) of 0.05. Each colored dot represents a differentially abundant protein fulfilling the significance criteria.

Volcano plot analysis using the paired Welch *t*-tests resulted in the detection of 421 and 212 proteins significantly altered in abundance in the hp-MKTPC cell proteomes and secretomes, respectively (Figure 3a,b). A complete list of differentially abundant proteins for proteomes and secretomes can be found in Tables S3 and S4 (Supplementary Materials), respectively. The secretome of hp-MKTPCs showed a nearly fivefold increased number of proteins reduced in abundance (176) compared to proteins increased in abundance (36). For the hp-MKTPC proteome, where 174 proteins were less abundant and 247 more abundant, the disparity was less pronounced. Strikingly, proteins involved in smooth muscle activity such as CNN1, ACTA2, MYH11, and desmin (DES) were found to be less abundant in the hp-MKTPC proteome. Further proteins with decreased abundance were factors such as nuclear factor (NF)-κB (NFKB1) and elongation factor 1-alpha 1 (EEF1A1), while regulatory proteins such as EGF-like repeat and discoidin I-like domain-containing protein 3 (EDIL3), ubiquitin-conjugating enzyme E2 J2 (UBE2J2), and plasminogen activator inhibitor 1 (SERPINE1) were found to be more abundant in the proteome of passaged MKTPCs.

In order to examine how much abundance alterations in the secretomes were due to protein secretion rather than cell degradation, all differentially abundant proteins of the secretome were annotated for “extracellular region” (GO:0005576) and the UniProt keyword “secreted”. Accordingly, proteins secreted via classical secretory pathway and via nonclassical secretion were predicted by SignalP and SecretomeP, respectively. In total, 66 classically secreted proteins and 35 proteins with nonclassical secretion were found. Only 31 from 212 proteins had neither of the four annotations, indicating only minor contaminations of proteins released from dying cells. In the secretomes, several members of the collagen family and laminins, e.g., collagen alpha-1(VI) chain (COL6A1), collagen alpha-2(VI) chain (COL6A2), laminin subunit beta-1 (LAMB1), and laminin subunit gamma-1 (LAMC1), were found to be less abundant in hp-MKTPCs. Superoxide dismutases (SOD1, SOD2, and SOD3), peroxiredoxins (PRDX1, PRDX2, PRDX4, and PRDX6), and macrophage migration inhibitory factor (MIF) were also found to be less abundant. Calreticulin (CALR) showed one of the strongest declines in abundance (log_2_ fold change: −6.5). Prominent proteins with increased abundance in hp-MKTPC secretomes were regulatory proteins such as cadherin-2 (CDH2) and Ras-related protein Rab-1A (RAB1A).

**Figure 4:**
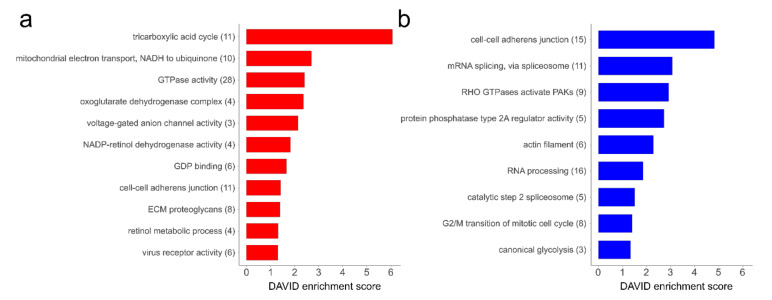
Enriched Database for Annotation, Visualization, and Integrated Discovery (DAVID) annotation clusters from proteins more abundant (**a**) and less abundant (**b**) in hp-MKTPCs. The number in brackets behind the term indicates the number of proteins in one cluster. Results with an enrichment score >1.3 were considered significant. Categories used for the DAVID tool were as follows: Gene Ontology (GO) biological process, GO cellular component, GO molecular function, and reactome. NADH: nicotinamide-adenine dinucleotide, reduced, GDP: guanosine diphosphate PAK: p21 activated kinase

To obtain deeper insight into the biochemical pathways related to the observed changes, a DAVID annotation cluster analysis of the proteins, which were altered in the proteomes, was performed. This resulted in 20 enriched functional clusters (Figure 4). In the group of proteins more abundant in hp-MKTPCs, different metabolic processes, including the mitochondrial tricarboxylic acid cycle, reactions involving GTPases, and endoplasmic-reticulum-related terms such as retinol metabolic process, as well as extracellular matrix proteoglycans, were significantly enriched (Figure 4a). In contrast, proteins related to glycolysis, cell cycle, RNA, and splicing processes were found to be enriched in the set of proteins less abundant in hp-MKTPCs (Figure 4b). Additionally, actins, proteins binding to actin filaments, and proteins related to the term “cell–cell adherens junction” were found to be significantly enriched in the datasets of both up- and downregulated proteins.

### 3.2. Proteome Analysis of Cells and Secretomes of MKTPCs from Young and Old Donors

**Figure 5:**
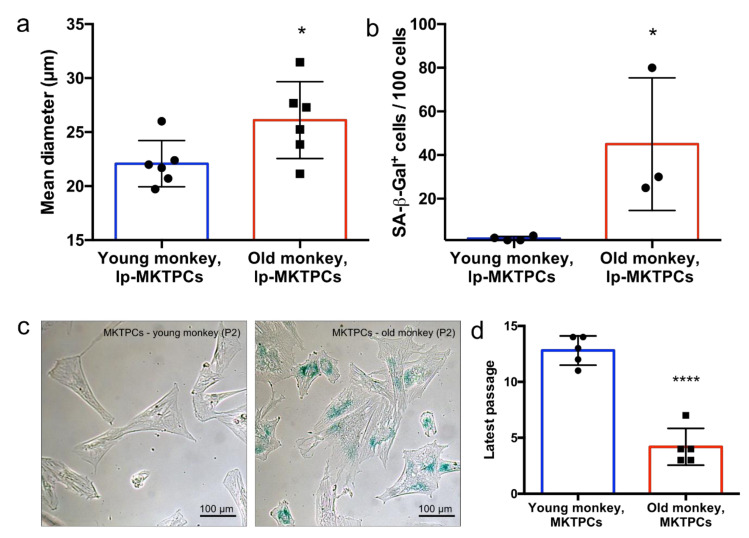
(**a**) Cells size measurement of MKTPCs from young vs. old monkeys revealed significantly increased cell size of MKTPCs from older monkeys. (**b**) Proportion of β-galactosidase-positive MKTPCs from young vs. old monkeys. (**c**) Light micrograph of senescence-associated β-galactosidase staining of MKTPCs from young (3 years) and old (11 years) monkey in passage 2. (**d**) Maximal passage numbers before offset of cell division. Columns indicate the mean; bars indicate the standard deviation. For statistical analysis, unpaired *t*-tests were used (**a**,**b**,**d**). * *p*-value < 0.05; **** *p*-value < 0.00005

**Figure 6:**
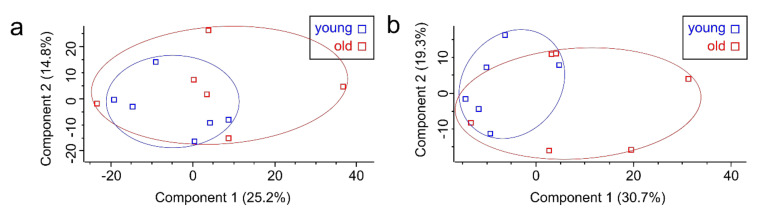
Principal component analysis of cell proteomes (**a**) and secretomes (**b**) of MKTPCs derived from young and older individual donors.

To further investigate aging-related changes in TPCs, we compared MKTPCs from six young and six old *C. jacchus* individuals. Young donors were either 2 or 3 years old, while the old donors were between 9 and 15 years old. The MKTPCs from older donors showed a significantly increased cell size (Figure 5a) and expressed a significantly increased amount of senescence-associated beta galactosidase (Figure 5b,c). MKTPCs from young individuals entered cell-cycle arrest and showed clear signs of senescence after 11–14 passages, while only 3–7 passages were needed to trigger senescence of MKTPCs from old animals (Figure 5d). The proteome and the secretome of MKTPCs from young and old donors were analyzed and led to the identification of 4534 and 1192 proteins with 51,801 and 9729 peptides, respectively (Table S5 and S6, Supplementary Materials). However, in contrast to the lp-MKTPC vs. hp-MKTPC comparison, the principal component analysis showed no clear separation between TPC proteomes and secretomes from young and old individuals (Figure 6). Strikingly, the quantitative protein profiles of the older individuals showed higher variation in components one and two than the protein profiles of the young individuals.

**Figure 7:**
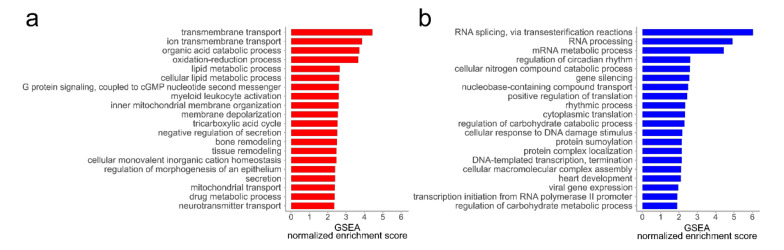
The 20 most significant gene set hits of MKTPC proteomes from older vs. young individual donors. Significant Gene Set Enrichment Analysis (GSEA) results (FDR q-value < 0.05) were summarized by reducing redundancy with REVIGO. Only gene sets from the GO biological process category were utilized. Gene sets enriched in the MKTPC proteome of old donors (**a**) and enriched gene sets in the MKTPC proteome of young donors (**b**) are displayed.

The statistical evaluation of the data using unpaired Welch modified *t*-tests did not lead to the detection of proteins significantly (FDR < 5%) altered in abundance. A Volcano plot analysis also did not show any significant abundance alterations (Figure 7a,b) after correction for multiple testing (FDR < 5%). However, while the cell proteome plot was quite symmetrical, the plot of secretomes was skewed toward negative log_2_ fold changes. This indicates a reduced secretion activity of MKTPCs of old animals, for which *p*-values of individual proteins were too high to be considered as significant after *p*-value correction for multiple testing.

To perform a statistical analysis at the level of related functional terms rather than at the level of individual proteins, a Gene Set Enrichment Analysis (GSEA) was performed. While the analysis of the secretome data resulted in only a small amount of regulated gene sets (data not shown), the MKPTC proteome analysis resulted in 560 enriched gene sets at an FDR < 0.05 (351 increased and 209 decreased, Tables S7 and S8, Supplementary Materials). The most prominent gene sets within the proteins more abundant in samples from old animals were related to metabolic processes such as the tricarboxylic acid cycle (TCA) and lipid metabolic processes, but also contained terms related to membrane and secretion (Figure 7a,b). Enriched terms from the set of downregulated proteins contained RNA- and DNA-centered processes and, to a lesser extent, terms of metabolic processes related to nitrogen and carbohydrate compounds.

## 4. Discussion

### 4.1. General Remarks

In the present study, we compared MKTPCs derived from young donors of high (hp-MKTPC) and low passages (lp-MKTPC). As this in vitro model may not exactly reflect in vivo aging, we further analyzed MKTPCs from young donors with MKTPCs from old donors (9–15 years). As captive marmosets show signs of aging already at the age of 8 years, the animals in the older group can be considered aged or even old [33,46,47]. The comparison of data from both sample sets enabled us to clarify to what extent repeated passaging reflects in vivo TPC aging. We focused on the analysis of TPC proteomes using a straightforward label-free mass spectrometry-based approach and detected over 5000 proteins. This high number of identified proteins corresponds to the analytical depth achieved by recent proteomic studies in the testes of other species [48,49,50]. Among the identified proteins, we found MYH11, ACTA2, and CNN1, all related to the typical smooth muscle-like phenotype of TPCs. Furthermore, we detected low abundant factors, e.g., NF-κB, demonstrating the sensitivity of our approach. In addition to cell lysates, we analyzed conditioned cell culture media to further monitor age-related alterations of the TPCs secretory activity. Out of these secretomes, we were able to identify nearly 1200 proteins, among them low abundant factors such as the important immune signaling factor MIF, demonstrating the analytical depth and biological relevance of our dataset.

### 4.2. Alterations in the Proteome of hp-MKTPCs Point to Impaired Signaling, Reduced Contractility, and Altered RNA Processing in Senescent MKTPCs

Among the proteins more abundant in hp-MKTPCs compared to lp-MKTPCs, several are related to mitochondrial energy production via the TCA cycle and the electron transport chain (Figure 4a). In contrast, senescent HTPCs showed a decrease in mitochondrial proteins and a degrading mitochondrial ultrastructure [30]. This is in line with mitochondrial dysfunction described as a classic hallmark of senescence [1,51]. Nevertheless, since only a small subclass of mitochondrial proteins were found to be altered in abundance in hp-MKTPCs, monkey TPC mitochondria may have been less affected by passaging than their human counterparts.

Many other findings in hp-MKTPCs, e.g., the increased abundance of Ras-related and GDP-binding proteins (Figure 4a), are in line with the results of senescent HTPCs [30]. In detail, we found the Ras-related proteins Rab-1A, Rab-21, Rab-5B, Rab-32, Rab-31, Rab-5C, and Rab-5A more abundant in hp-MKTPC. Ras-related proteins are small GTPases which regulate vesicle formation and membrane trafficking and can have a variety of functions [52]. For example, Ras-related protein Rab-1A (RAB1A) is involved not only in interleukin (IL)-8 and growth hormone secretion but also in autophagy and cell migration [53,54]. Furthermore, many Ras-related proteins are known to act as oncogenes and tumor-suppressive regulators [55]. Considering the broad role of this protein class in disease and male fertility, alterations in their abundance may reflect higher risks of tumorigenesis and an impaired spermatogenesis of the aging testis [56,57].

The functional enrichment analysis of the proteins with higher abundance in hp-MKTPCs revealed an overrepresentation of extracellular matrix (ECM) proteins associated with the reactome term “ECM proteoglycans”. Among them, we found the transmembrane receptors integrin alpha-2 (ITGA2) and integrin beta-5 (ITGB5), as well as dystroglycan (DAG1) and laminin subunit beta-1 (LAMB1). The two latter proteins are known to be localized at the basement membrane. In addition to its structural functions and its close proximity to TPCs, the basement membrane further acts as a platform for complex signaling and is an important player for extracellular matrix organization.

Since the maintenance of the ECM is a key feature of TPCs, the observed abundance alterations may reflect irregularities in this task. Additionally, we found an increased abundance of SERPINE1 in hp-MKTPCs, which is a downstream target of p53-triggered senescence [58]. Together with the alterations in Ras-related proteins, all these findings clearly indicate impairments in hp-MTKPCs signaling. This finding is further supported by a decreased abundance of the p105 subunit of NF-κB (NFKB1) in hp-MKTPC. NF-κB is a pleiotropic transcription factor which is regulated by various signal transduction pathways and fulfills roles in various biological processes. Interestingly, NFKB1- deficient mice show an early onset of aging [59]. From the set of proteins less abundant in hp-MKTPCs, we found proteins related to the Gene Ontology terms “RNA processing” and “catalytic step 2 spliceosome” less abundant in hp-MKTPCs. An age-related downregulation of alternative splicing has already been described and was also found in HTPCs of high passage number [30,60,61]. In this context, two proteins are particularly interesting: heterogeneous nuclear ribonucleoprotein D0 (HNRNPD) and serine/arginine-rich splicing factor 7 (SRSF7). In a recent study, HNRNPD was knocked down in human endothelial cells, which resulted in an increased occurrence of senescence [62]. Furthermore, SRFSF7 is known to play a role in the regulation of alternative splicing of p53 and in the regulation of senescence [63]. These findings strongly suggest that RNA-binding proteins and alternative splicing play an important role in the development of senescence in TPCs.

In addition to changes related to RNA processing and splicing, the functional enrichment analysis revealed a significantly high proportion of proteins of the reactome pathway “Rho GTPases activate PAKs” (Figure 4b). This pathway is mainly involved in cytoskeletal reorganization and contains several proteins characteristic for TPCs such as myosin-10 (MYH10), myosin-11 (MYH11), and calmodulin-1 (CALM1), all found less abundant in hp-MKTPC. The pathway also includes myosin regulatory light chain 12B (MYL12B) and smooth muscle myosin light chain kinase (MYLK). Both regulate smooth muscle cell contraction and were less abundant. Proteins which are not part of this pathway but are also essential for contractility such as smooth muscle actin (ACTA2), desmin (DES), and calponin-1 (CNN1) were also less abundant in hp-MKTPCs. ACTA2, CNN1, and MYH11 are commonly described as contractility markers and are known to be reduced in patients with impaired spermatogenesis [10,34]. Furthermore, a decrease in DES-positive cells in elderly men’s testes was reported, making it a clear indicator of testicular aging [64]. Overall, the broad decrease in various structural and regulatory proteins points toward a reduced contractility. Contrary to the study in the human system [30], we provide clear evidence for the decreased abundance of key contractility markers.

### 4.3. The Secretome of hp-MKTPCs Indicates Alteration in Reactive Oxygen Species (ROS) Handling and Signaling

Strikingly, similar to the proteomes, many alterations in abundances found in hp-MKTPC secretomes were previously found in HTPCs, which underlines the relevance of our nonhuman primate model for the human system. For instance, a lower abundance of different alpha collagens and laminins was found in hp-MKPTCs, highlighting the impact of TPC senescence on the ECM (Figure 3b) [30]. In contrast to the HTPC results, macrophage migration inhibitory factor (MIF) was found in lower abundance in hp-MKTPCs, while it was more abundant in senescent HTPC secretomes. Since MIF is a proinflammatory cytokine which can activate NF-κB, this could indicate a different role for immune signaling in the young MKTPC donors compared to the aged HTPC donors [65]. Another protein involved in NF-κB signaling is the calcium-binding chaperone calreticulin (CALR). It is one of the proteins with the most reduced abundance (log_2_ fold change: −6.5) in hp-MKTPC secretomes. With respect to the regulation of CDK-Inhibitor 1 (p21), CALR competes with CUGBP Elav-like family member 1 (CELF1), which was found to be more abundant in the hp-MKTPC proteome [66]. The binding of CELF1 instead of CALR to p21 RNA leads to growth arrest and, consequently, to senescence. Therefore, the observed change in the ratio of CALR/CELF1 may reflect a reduced CALR repression of p21 translation and clearly indicates an involvement of p21 in the cellular senescence of MKTPCs.

Among the proteins less abundant in hp-MKTPC secretomes were several peroxiredoxins and superoxide dismutases, all involved in the degradation of reactive oxygen species (ROS). This is in line with results from several studies which support the idea that increased ROS is not necessarily the cause, but the result of aging and possibly caused by a reduced abundance of ROS handling enzymes in senescent cells [67]. However, it is known that a knockdown of Cu–Zn superoxide dismutase (SOD1) leads to senescence, which supports the common hypothesis that excessive ROS triggers senescence [68,69]. Even though this study cannot resolve if ROS are the product or the inducer of senescence in hp-MKTPCs, it emphasizes the role of ROS in senescent MKTPCs.

Strikingly, in hp-MKTPC secretomes, we found a considerably higher number of proteins decreased rather than increased in abundance compared to lp-MKTPC. This hints to a lower capability of senescent cells to secrete proteins. Furthermore, cadherin-2 (CDH2), a protein important for cell–cell adhesion, and Ras-related protein Rab-1A (RAB1), which was also more abundant in hp-MKTPC proteomes, were the two proteins with the strongest increase in abundance in hp-MKTPC secretomes [70]. These proteins—so far not reported in the context of cellular senescence—illustrate that senescence-induced protein secretion may also influence other cell types in the testis. Several proteins with decreased abundance in the secretome of hp-MKTPCs, i.e., fibronectin, laminins, collagens, and MIF, also imply a senescence-associated secretory phenotype (SASP) affecting testicular signaling [25,71].

### 4.4. Proteomics Reveals Reduced Contractility Markers and Impaired Secretion as Subtle Signs of Senescence in the Proteome of Older MKTPCs

**Figure A1:**
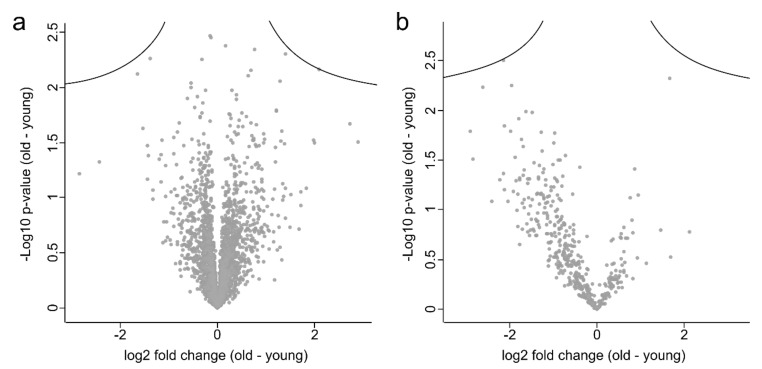
Volcano plot analysis of proteomes (**a**) and secretomes (**b**) of MKTPCs from older and younger individual donors. Proteins above the significance cutoff curve (s = 0.1; FDR < 0.05) are considered significant.

Like hp-MKTPCs, MKTPCs from old donors showed characteristic hallmarks of senescence, namely, beta galactosidase expression and increased cell size (Figure 5). However, in contrast to the passaged MKTPCs, PCA did not lead to a clear separation of proteomes and secretomes of MKTPCs from old and young donors (Figure 6). Additionally, neither statistical Welch’s *t*-tests nor volcano plot analyses revealed differentially abundant proteins (Figure A1, Appendix A). Thus, alterations in the old MKTPC group were less pronounced than in in vitro induced senescence in hp-MKTPC.

In contrast to their young counterparts, the proteome and secretome profiles from the old MKTPC group showed higher variations, which may be related to the fact that this group was composed of donors with an age range of 9–15 years and, therefore, somewhat heterogeneous. Additionally, similar to the passaged MKTPCs, the asymmetrical volcano plot of the secretome suggests a reduced secretory activity of MKTPCs from old animals as compared to young animals. In combination with the fact that MKTPCs of old individuals could be significantly less often passaged and showed other clear signs of senescence, these observations suggest differences in the corresponding cell proteomes. Indeed, a Gene Set Enrichment Analysis comparing datasets of older vs. young MKTPCs led to several significantly enriched gene sets in the cell proteome (Figure 7).

Similar to the results from the passaged MKTPCs, several enriched gene sets are related to metabolic pathways, especially mitochondrial pathways such as the TCA cycle, which was the most enriched pathway in hp-MKTPCs. Particularly interesting is the enrichment of proteins related to the term “negative regulation of secretion”, which fits the decreased secretion observed for many proteins. The set of proteins less abundant in older MKTPC showed an enrichment for various terms related to RNA processing and splicing, which was also detected in hp-MKTPCs and was discussed as a sign of aging and senescence (see above). Furthermore, proteins related to smooth muscle cell contraction were found in the terms “heart development” and in the reactome term “smooth muscle contraction”, both significantly decreased (FDR < 0.05), again indicating a possible loss of contractility. Incidentally, three of the enriched proteins in the downregulated term “regulation of circadian rhythm” were non-POU domain-containing octamer-binding protein (NONO), splicing factor, proline- and glutamine-rich (SFPQ), and paraspeckle component 1 (PSPC1). All three proteins were previously reported to be expressed in Sertoli cells and play a role in androgen receptor signaling [72]. The androgen receptor also plays an active role in TPCs which makes these three proteins an interesting new target for follow-up studies to explore their specific roles in TPCs [73]. Two negatively enriched gene sets were reactome “cellular senescence” and “cell cycle”. One prominent member of these enriched terms was again NF-κB, which was already found significantly lower abundant in senescent MKTPCs and HTPCs, indicating again alterations in the immune signaling of senescent TPCs.

## 5. Conclusions

This study demonstrates that replicative senescence in hp-MKTPCs is associated with a variety of alterations in the proteome and secretome. The detected alterations showed a high degree of similarity to results previously obtained in human TPC, which demonstrates that the nonhuman *C. jacchus* model reliably reflects the human system [30]. The detected proteome and secretome alterations in senescent MKTPCs strongly suggest impairments of protein secretion and ECM modulation, as well as a decreased capacity to handle ROS. Furthermore, our results provide evidence for changes in RNA processing and alternative splicing, for NF-κB-modulated immune signaling, and for a reduced capability of senescent MKTPCs to contract.

In addition to studies on TPC senescence induced by repeated cell passaging, the common marmoset model facilitates studies and comparisons of young and older TPCs aged in vivo. Even though the alterations within the proteomes and secretomes of in vivo aged MKTPCs are less pronounced than in in vitro aged TPCs, we again found evidence for an impaired protein secretion, for alterations in splicing, and for a reduced contractility of in vivo aged MKTPCs. These findings demonstrate the involvement of TPCs in testicular aging.

However, it has to be considered that, even though proteomics is a powerful research tool, facilitating the quantification of thousands of proteins, proteome alterations alone cannot completely characterize the entire mechanism of a complex process such as cellular aging. Nevertheless, the detected senescence-related proteome alterations and the associated biochemical pathways are particularly valuable and can serve as a basis for future functional and mechanistic experiments dedicated to improving the understanding of cellular aging.

## Supplementary Materials

The following data are available online at https://www.mdpi.com/2073-4409/9/11/2498/s1: Table S1: All proteins identified in the proteomes lp- and hp-MKTPCs; Table S2: All proteins identified in the secretomes lp- and hp-MKTPCs; Table S3: Proteins significantly different in abundance in hp-MKTPC proteomes (paired Welch *t*-test, q-value < 0.05) between low and high passages of MTPCs; Table S4: Proteins significantly different in abundance in hp-MKTPC secretomes versus lp-MKTPCs secretomes; Table S5: All proteins identified in the proteomes of young and old MKTPCs; Table S6: All proteins identified in the secretomes of young and old MKTPCs; Table S7: GSEA results: gene sets enriched in older MKTPCs; Table S8: GSEA results: gene sets enriched in older MKTPCs.
